# Efectos adversos de las sustancias modelantes en Cali, Colombia

**DOI:** 10.7705/biomedica.5707

**Published:** 2020-10-16

**Authors:** Claudia Marcela Castro, Carlos Alberto Ríos, Carlos Alejandro López, Martha Lucía Ospina, Yamileth Ortiz

**Affiliations:** 1 Equipo Banco de Proyectos, Dirección de Investigación en Salud Pública, Instituto Nacional de Salud, Bogotá, D.C., Colombia; 2 Centro Médico Santuario, Cali, Colombia; 3 Dirección General, Instituto Nacional de Salud, Bogotá, D.C., Colombia; 4 Dirección de Investigación en Salud Pública, Instituto Nacional de Salud, Bogotá, D.C., Colombia

**Keywords:** biopolímeros, efectos adversos, enfermedad iatrogénica, contraindicaciones de los procedimientos, estética, procedimientos quirúrgicos reconstructivos, Biopolymers, adverse effects, iatrogenic disease, contraindications, procedure, esthetics, reconstructive surgical procedures

## Abstract

**Introducción:**

El deseo de mejorar la apariencia física mediante métodos sencillos y económicos, ha generado la aplicación indiscriminada de sustancias modelantes y, con ello, el surgimiento de la alogenosis iatrogénica, enfermedad cada vez más prevalente en Latinoamérica.

**Objetivo:**

Describir las características epidemiológicas y los efectos adversos de las sustancias modelantes en un grupo de pacientes de Cali, Colombia.

**Materiales y métodos:**

Se hizo una revisión retrospectiva de las historias clínicas de los pacientes que acudieron a consulta por complicaciones producidas por sustancias modelantes durante un sexenio.

**Resultados:**

Se incluyeron 1.322 pacientes, 95,5% de ellos mujeres. Las edades oscilaron entre los 19 y los 83 años, con una media de 39 años. El sitio anatómico de infiltración con sustancias modelantes con mayor frecuencia de efectos adversos, fueron los glúteos. La asimetría y el aumento del volumen en el sitio infiltrado fueron los signos más comunes, en tanto que el dolor, las alteraciones del ánimo y la depresión o la ansiedad fueron los síntomas más percibidos. El 33,6% de los pacientes desconocía la sustancia aplicada y el 28,1% refirió haberse aplicado biopolímeros. La mayoría de estos procedimientos estuvo a cargo de personal sin la debida formación.

**Conclusiones:**

Estos pacientes requieren la atención de equipos multidisciplinarios para establecer alternativas de tratamiento que mejoren su calidad de vida. Además, se necesitan la regulación de los establecimientos, y las medidas de vigilancia, inspección y control en la importación y el uso de estas sustancias.

## Abstract (en)

**Introduction:**

The desire to improve one’s physical appearance through simple and economical methods has resulted in the indiscriminate application of modeling substances. As a result, iatrogenic allogenosis has emerged as an increasingly prevalent disease in Latin America.

**Objective:**

To describe the epidemiological characteristics and adverse effects arising from the use of modeling substances in a group of patients from Cali, Colombia.

**Materials and methods:**

We conducted a retrospective review of the medical records of patients who consulted for complications arising from the use of modeling substances during a six-year period.

**Results:**

A total of 1,322 patients were included of whom 95.5% were women. Patients’ ages ranged from 19 to 83 years, with an average of 39 years. The most infiltrated anatomical site showing adverse effects due to modeling substances was the buttocks. The asymmetry and increased volume of the infiltrated site were the most common signs while pain, mood disturbances, and depression or anxiety were the most commonly perceived symptoms. A total of 41.8% of patients ignored what substances they had received, and 28.5% received biopolymers; these procedures were mostly performed by non-qualified personnel.

**Conclusions:**

The care of these patients requires multidisciplinary teams to establish treatment alternatives to improve their quality of life. In addition, the regulation of establishments, as well as the surveillance, inspection, and control of the imports and use of these substances should be warranted.

Durante los últimos años se ha observado un incremento en la utilización de sustancias modelantes para mejorar la apariencia física bajo la premisa de que son métodos sencillos, poco dolorosos y económicos, pero desconociendo las posibles consecuencias y complicaciones (1).

Según la última encuesta de la Sociedad Internacional de Cirugía Plástica Estética, durante el 2018 los tratamientos inyectables representaron el 76% de los procedimientos no quirúrgicos en los 10 países con mayor número de procedimientos estéticos a nivel global, lo que representa un cambio en las tendencias, ya que las intervenciones no quirúrgicas se han convertido en una opción para quienes desean modificar su apariencia con resultados inmediatos después de la inyección (2).

Con el propósito de modelar el contorno corporal, personal experto e inexperto inyecta indiscriminadamente fluidos de alta densidad o sustancias modelantes, clasificadas como sustancias reabsorbibles (ácido hialurónico, ácido poli L-láctico, fosfato tricálcico y el polisacárido recubierto de alginato) o no absorbibles (silicona, poliaquilamida, poliacrilamida, polimetilmetacrilato) y otras sustancias, como aceites vegetales, aceite de motor, cera de abeja y grasa animal (3-8). Ello ha generado una nueva enfermedad conocida como enfermedad por modelantes o alogenosis iatrogénica (9), cuyas consecuencias se están convirtiendo en un problema emergente de salud, principalmente en los países de Latinoamérica.

Las consecuencias de su uso involucran daños físicos, mentales y económicos. Las reacciones adversas más comunes comprenden formaciones quísticas con riesgo potencial de infección y abscesos, formación de granulomas, adelgazamiento de la piel con cambios tróficos como esclerosis, hipopigmentación o hiperpigmentación, fístulas y manifestaciones más graves, como la necrosis progresiva hacia la profundidad del tejido. Aunque el porcentaje de casos que han terminado con sepsis grave, discapacidad o muerte es bajo, estas complicaciones ya están siendo documentadas (1,5,10). Se han descrito tres momentos en la historia natural de la aplicación de los modelantes: la muerte inmediata después de su aplicación, signos de irritación al poco tiempo de la inyección, y síntomas tardíos que pueden aparecer después de muchos años; al parecer, en algunos casos se producen efectos secundarios no visibles (11).

Según Martínez, *et al.*, Brasil, Argentina, Venezuela, Colombia y México lideran las cifras epidémicas a nivel mundial. En dos estudios recientes en Colombia, se reportó el desarrollo de este síndrome en el curso de 10 a 18 años (9,11), pero en el país no existen cifras oficiales que cuantifiquen este problema; solo en algunos estudios se han aportado datos comprobados que deben servir para sensibilizar a los responsables de las decisiones sobre las medidas que deberían ser adoptadas.

El propósito de artículo fue describir las características epidemiológicas y los efectos adversos derivados de la inyección de sustancias modelantes en un grupo de pacientes en Cali.

## Materiales y métodos

### Diseño

Se hizo un estudio observacional retrospectivo en el Centro Médico Santuario de Cali para el periodo de 2013 a 2018. Se revisaron las historias clínicas de los pacientes que consultaron por reacciones adversas derivadas de la inyección de sustancias modelantes, de cualquier edad, sexo y lugar de procedencia, siempre que estas variables no presentaran pérdida de información. El cuestionario para la recolección de datos incluyó variables demográficas (edad, sexo, lugar de residencia), clínicas (síntomas, signos) y las relacionadas con el contexto de aplicación de la sustancia. Los datos fueron consolidados en Excel y procesados en el programa *Statistical Package for the Social Sciences™* (SPSS), versión 12. Se hizo un análisis descriptivo utilizando medidas de frecuencia central, frecuencias absolutas y relativas.

### Consideraciones éticas

El proyecto obtuvo la aprobación del Comité de Ética y Metodologías de la Investigación del Instituto Nacional de Salud, código CTIN-21-2017.

## Resultados

Durante el período de estudio, 1.322 pacientes consultaron debido a efectos adversos o complicaciones por la inyección de una sustancia modelante, de los cuales el 95,5% correspondía a mujeres. Las edades oscilaron entre 19 y 83 años, con una media de 39 años (desviación estándar, DE=±12,2); el 61,9% de los pacientes tenía entre 30 y 49 años en el momento de la consulta (cuadro 1).

**Cuadro 1:** Características demográficas de los pacientes incluidos en el estudio. Centro Médico Santuario, Cali, 2013-2018

**Variable**	**Categoría**	**n**	**%**
Sexo	Femenino	1.262	95,5
	Masculino	57	4,3
Grupos de edad (años)	19	2	0,2
	20-29	232	17,5
	30-39	518	39,2
	40-49	290	21,9
	50-59	187	14,1
	60-69	62	4,7
	Mayor de 70	14	1,1
Lugar de procedencia	Valle del Cauca	935	70,7
	Cauca	38	2,9
	Antioquia	31	2,3
	Bogotá	24	1,8
	Otros departamentos	115	8,7
	Otros países	179	13,5

El 89,2% de los pacientes tenía un sitio anatómico infiltrado, el 9,8%, dos, y el 1,1%, tres. Los glúteos fueron el sitio anatómico más frecuentemente (74,4%) infiltrado, seguido por el rostro (19%) y, en menos del 1% de los casos, las áreas de infiltración fueron la región abdominal, el pene o los brazos.

En cuanto al lugar anatómico infiltrado, se encontraron diferencias por sexo, pues las mujeres presentaron mayor infiltración en glúteos y en regiones como las piernas y el abdomen, en tanto que, en los hombres, las infiltraciones fueron en el rostro y los brazos (cuadro 2).

**Cuadro 2:** Comportamiento por sexo y rango de edad según lugar anatómico infiltrado. Centro Médico Santuario. Cali, Colombia, 2013-2018

**Características demográficas**		**Porcentaje por lugar anatómico infiltrado**						
		**Glúteos**	**Rostro**	**Piernas**	**Senos**	**Abdomen**	**Pene**	**Brazos**
Sexo	Mujer	97,4	90,3	100	100	100	0	0
	Hombre	2,6	9,7	0	0	0	100	100
Rango de edad (años)	19	0,2	0	0	0	14,3	0	0
	20-29	20,2	8,7	10,4	14,3	28,6*	0	0
	30-39	44,4*	17,0	18,8	40,0	28,6	50,0	100
	40-49	22,1	21,0	27,1	28,6*	0	16,7	0
	50-59	10,7	33,0	41,7*	17,1	28,6	16,7	0
	60-69	2,1	15,9*	2,1	0	0	16,7	0
	>70	0,4	4,3	0	0	0	0	0

* Lugar anatómico infiltrado más común por rango de edad

Con relación a los rangos de edad, el 44,4% de los pacientes con infiltración en los glúteos tenía entre 30 y 39 años en el momento de la consulta, seguido por el grupo de 40 a 49 años. En los casos de infiltraciones en el rostro, uno de cada tres pacientes tenía entre 50 y 59 años, y en este mismo rango de edad, la mayoría de las infiltraciones se presentó en las piernas. En los adultos jóvenes, de 30 a 39 años, además de los glúteos, las infiltraciones se hicieron en regiones anatómicas como los senos y el pene. El 14,3% de los pacientes con infiltración en el abdomen tenía 19 años (cuadro 2).

El 41,8% de los pacientes desconocía la sustancia aplicada, el 28,5% refirió que se trataba de biopolímeros, el 14%, de ácido hialurónico, el 7,1%, de otras sustancias como aceites, grasa animal, vitamina C o plasma, el 3,7%, de polimetacrilato, el 2,4%, de silicona, y el 2,4%, de colágeno. En cuanto a las sustancias infiltradas en glúteos, se encontró que en el 50% de los casos se trataba de biopolímeros, en el 28%, ácido hialurónico, en el 18,5%, otras sustancias, y en el 7,6%, polimetacrilato.

Durante la primera consulta, los pacientes reportaron más de un signo y un síntoma debido al efecto adverso producido por la aplicación de la sustancia modelante, siendo la asimetría del lugar y el aumento de su volumen los signos más frecuentemente detectados, en tanto que el dolor, las alteraciones del ánimo y la depresión o ansiedad son los síntomas más percibidos (cuadro 3).

**Cuadro 3:** Signos y síntomas más comunes presentados por los pacientes. Centro Médico Santuario, Cali, 2013-2018

**Signos**	**n**	**%**
Asimetría	1.183	89,5
Aumento de volumen del lugar inyectado	935	70,7
Eritema	804	60,8
Hiperpigmentación	740	56,0
Cambio en el contorno del implante inyectado (deformidad)	736	55,7
**Síntoma**	**n**	**%**
Dolor general	865	65,4
Alteraciones en el estado de ánimo	766	57,9
Dolores musculares	680	51,4
Depresión o ansiedad	667	50,5
Dolores articulares	613	46,4

Según la información de la historia clínica, el 88,0% de los pacientes refirió antecedentes quirúrgicos por diferentes causas. El 7,7% presentaba migración de la sustancia infiltrada y, de este porcentaje, en el 33% se trataba de biopolímeros y en el 13%, de ácido hialurónico.

**Figura 1:**
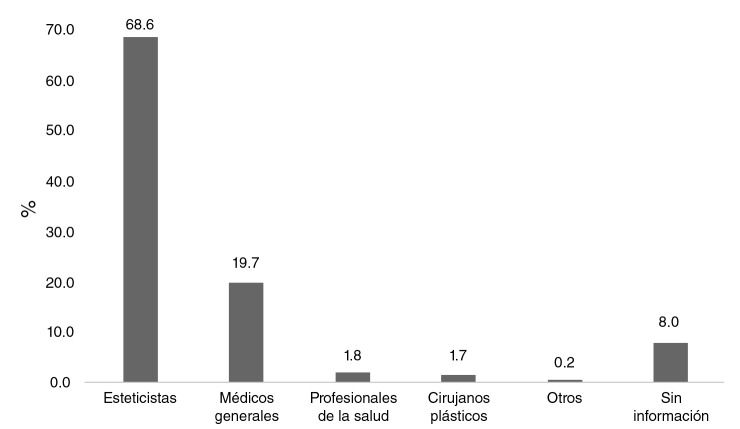
Personal referido por los pacientes como responsables de la aplicación de la sustancia modelante. Centro Médico Santuario, Cali, 2013-2018

Los pacientes refirieron que la mayoría de los procedimientos habían sido realizados, en primer lugar, por esteticistas, seguidos de médicos generales, otros profesionales de la salud como odontólogos, enfermeras y fisioterapeutas, médicos cirujanos plásticos y otros, incluidos abogados y tatuadores (figura 1).

Al indagar por el lugar del procedimiento, en el 65,2% de los casos se había hecho en centros de estética, en el 11,5%, en el sitio de residencia de la persona que aplicó la sustancia, en el 9%, en consultorios o clínicas, y en el 4,5%, en el domicilio del paciente.

## Discusión

Las sustancias modelantes se usan en hombres y mujeres de todas las edades con el fin de mejorar su apariencia física. Según los resultados de este estudio, la mayoría de los pacientes tenía edades entre los 30 y los 49 años en el momento de la consulta por un efecto adverso; sin embargo, los grupos de edad según las regiones infiltradas se determinaron a partir del momento en que se manifestó dicho efecto, lo cual no es lo mismo que el tiempo de depósito o migración de la sustancia en el organismo. La infiltración de glúteos fue la más común en este rango de edad, en tanto que, en los mayores de 60 años, fue la facial.

En México, Martínez, *et al.* (3), reportaron, entre los sitios anatómicos más afectados, los glúteos (38,5%), las piernas (18%), los muslos (15,4%) y los senos (11,8%). En Cali, la infiltración de las piernas y los senos no fue tan prevalente, lo cual podría explicarse por las diferencias en el número de pacientes incluidos en los estudios o por los factores culturales de los países.

El 10,9% de los pacientes en este estudio se había sometido a más de un procedimiento de infiltración en lugares anatómicos diferentes, lo cual supone una condición agravada que representa un desafío para el cirujano, dado que, en la mayoría de los casos, deben hacerse varias cirugías. Entre las técnicas de manejo terapéutico que buscan brindar soluciones al paciente, solo con la resección y el retiro de la sustancia infiltrada se han reportado buenos resultados, aunque ello acarrea deformidades y la necesidad de reconstrucción de los defectos resultantes (5).

Las sustancias modelantes infiltradas en el cuerpo deberían ser seguras, biocompatibles, no deberían tener efectos inmunogénicos, carcinogénicos, teratogénicos o infecciosos, no deberían migrar y deberían ser de fácil extracción (12,13). En este estudio, el 36% de los pacientes se había sometido a inyecciones de sustancias ilegales, como biopolímeros y aceites, y el 28% de aquellos con infiltración en los glúteos manifestó haberse inyectado ácido hialurónico. La aplicación de grandes volúmenes de ácido hialurónico específicamente en los glúteos, sería inviable económicamente por su alto costo, por lo que se supone que lo que realmente se aplica es otra sustancia ilícita diferente (11). Los estudios de seguridad del ácido hialurónico no han evidenciado efectos secundarios, solo se ha reportado la formación de nódulos por la aplicación superficial del producto y, en pocos casos, la aparición tardía de reacciones granulomatosas, atribuidas a una posible reacción alérgica o a una reacción a cuerpo extraño, así como de infección, absceso estéril o biopelícula (14).

Es importante que el personal que aplica la sustancia modelante tenga una comprensión cabal de la anatomía humana, que seleccione adecuadamente los pacientes y los productos, y que tenga conocimiento de las técnicas correctas de preparación e inyección. Además, en todo caso, sería preferible que dichos procedimientos fueran realizados por un cirujano plástico (15).

En cuanto a los signos y síntomas, la asimetría, el aumento de volumen y el dolor reportados en el presente estudio también lo fueron en México; las infecciones solo se presentaron en el 2,6%, porcentaje inferior al 11,7 reportado en México en un estudio en que se encontraron *Mycobacterium fortuitum* y *Escherichia coli* productora de beta-lactamasa de espectro extendido (BLEE) en las muestras (3).

Cabe destacar que el 88% de los pacientes presentaba antecedentes quirúrgicos por diferentes causas. En futuros estudios prospectivos, sería importante evaluar el tipo de procedimiento quirúrgico previo, así como el estado inmunológico de los pacientes para establecer una posible explicación o relación.

Se ha descrito que la inyección de sustancias modelantes como aceite mineral, ácido hialurónico, colágeno y silicona, puede inducir enfermedades del sistema inmunológico y, en algunos casos, el desarrollo del síndrome autoinmunitario-inflamatorio inducido por adyuvantes, con síntomas como artralgia y fatiga crónica asociados con un proceso inflamatorio crónico (16,17).

Cerca del 50% de los pacientes reportaron alteraciones en el estado de ánimo y depresión pues, como ya se mencionó, esta problemática va más allá del aspecto físico e involucra aspectos psicológicos y económicos que pueden conllevar gastos catastróficos en salud. Su impacto social, además, llega a los medios de comunicación, en tanto que sus consecuencias económicas y legales comprometen el sistema de salud por sus altos costos. No obstante, aunque en la mayoría de los casos la reparación quirúrgica es asumida por el paciente, cuando la asumen las empresas prestadoras de servicios de salud, el gasto de bolsillo no se ha calculado (11).

En nuestro estudio, la migración de la sustancia hacia sitios lejanos del sitio inyectado se presentó en el 7,7% con consecuencias y secuelas dolorosas que, en algunos casos, no era posible tratar. Esta migración depende de la viscosidad de la sustancia y el incremento en la presión local ante la aplicación de masajes, y puede presentarse, incluso, entre tres y 15 años después de su aplicación (18,19).

Dado que este es un procedimiento ilegal, podría haberse esperado que los encargados de hacerlo no incluyeran a personal de la salud; sin embargo, el 20% de las infiltraciones estuvo a cargo de médicos, incluidos especialistas como dermatólogos o cirujanos plásticos. Este fenómeno estaría relacionado con el deseo de lucro, el desconocimiento de los efectos adversos de su aplicación o la utilización de sustancias ilícitas de dudosa procedencia adquiridas a muy bajo precio (14).

La falta de regulación en el país para el uso de estas sustancias, sumado al poco control y vigilancia de los centros de estética, se correlaciona con los resultados de este estudio, pues más del 65% de los procedimientos se llevó a cabo en estos establecimientos. En este sentido, Cala-Uribe, *et al.*, reportaron varios casos de mortalidad y lesiones personales en el país debidos a la infiltración de materiales extraños, después de revisar expedientes judiciales de la Fiscalía General de la Nación (20).

Ante los vacíos en el conocimiento del tema, es necesario que este sea abordado por grupos multidisciplinarios que puedan dar respuestas a los pacientes sobre alternativas de tratamiento y mejoramiento de su calidad de vida, además de las necesarias regulación y adopción de políticas de vigilancia y control.
